# Treatment of Acquired Hypothalamic Obesity: Now and the Future

**DOI:** 10.3389/fendo.2022.846880

**Published:** 2022-04-06

**Authors:** Paul Dimitri

**Affiliations:** ^1^The Department of Paediatric Endocrinology, Sheffield Children’s NHS Foundation Trust, Sheffield, United Kingdom; ^2^College of Health, Wellbeing and Life Sciences, Sheffield Hallam University, Sheffield, United Kingdom

**Keywords:** hypothalamic obesity, craniopharyngioma, suprasellar tumors, hypothalamus, insulin, GLP1, oxytocin, methionine aminopeptidase inhibitors

## Abstract

The hypothalamus is the centre of neuroendocrine regulation of energy homeostasis and appetite. Maldevelopment of, or damage to, the key hypothalamic nuclei disrupts the coordinated balance between energy intake and expenditure leading, to rapid and excessive weight gain. Hypothalamic obesity is compounded by a disruption of the hypothalamic-pituitary axis, sleep disruption, visual compromise, and neurological and vascular sequalae. Amongst suprasellar tumors, craniopharyngioma is the most common cause of acquired hypothalamic obesity, either directly or following surgical or radiotherapeutic intervention. At present, therapy is limited to strategies to manage obesity but with a modest and variable impact. Current approaches include optimizing pituitary hormone replacement, calorie restriction, increased energy expenditure through physical activity, behavioral interventions, pharmacotherapy and bariatric surgery. Current pharmacotherapeutic approaches include stimulants that increase energy consumption, anti-diabetic agents, hypothalamic–pituitary substitution therapy, octreotide, and methionine aminopeptidase 2 (MetAP2) inhibitors. Some pharmacological studies of hypothalamic obesity report weight loss or stabilization but reported intervention periods are short, and others report no effect. The impact of bariatric surgery on weight loss in hypothalamic obesity again is variable. Novel or combined approaches to manage hypothalamic obesity are thus required to achieve credible and sustained weight loss. Identifying etiological factors contributing hypothalamic obesity may lead to multi-faceted interventions targeting hyperphagia, insulin resistance, decreased energy expenditure, sleep disturbance, hypopituitarism and psychosocial morbidity. Placebo-controlled trials using current single, or combination therapies are required to determine the impact of therapeutic agents. A well-defined approach to defining the location of hypothalamic damage may support the use of future targeted therapies. Intranasal oxytocin is currently being investigated as an anorexogenic agent. Novel agents including those targeting pro-opimelanocortin-C and AgRP/NPY expressing neurons and the MC4 receptor may result in better outcomes. This article discusses the current challenges in the management of hypothalamic obesity in children and young people and future therapeutic approaches to increasing weight loss and quality of life in these patients.

## 1 Introduction

The potential for a lesion in the hypothalamic-pituitary region to result in obesity is not a recent discovery. Over 180 years ago, von Mohr published a case of rapid onset obesity in a case of a pituitary tumour (1), and in 1939, Nevin published a report relating to the sequalae of hypothalamic-pituitary tumours which included ‘adiposity that may precede the depression of sexual function’ (2). At around the same time, the concept that the hypothalamus was in some way associated with fat metabolism was emerging (3), and only 4 years later, Brobeck demonstrated that rats with lesions in the hypothalamic ventromedial nucleus (VMN) showed voracious hunger (4); 10 years later, the central role of the hypothalamus in energy regulation was first proposed (5). It is over the last few decades that our knowledge of the hypothalamus as a key regulator energy regulation and appetite has significantly increased.

### 1.1 Hypothalamic Control of Energy Regulation and Appetite

Nuclei in the mediobasal hypothalamus play a fundamental role in energy balance through the modulation of appetite and food consumption, regulation of fat storage and energy expenditure. By responding to circulating signals from peripheral energy stores the mediobasal hypothalamus adjusts energy consumption to ensure that body weight and in particular body fat remain stable (6, 7). The arcuate nucleus (ARC) within the mediobasal hypothalamus is considered the hypothalamic area that primarily senses metabolic signals from the periphery *via* the systemic circulation or the CSF, facilitated by its adjacency with the median eminence, and the third ventricle. There are two distinct populations of neurones within the (ARC) that balance food intake, through the production of the orexigenic peptides (appetite promoting) neuropeptide Y (NPY) and agouti-related peptide (AgRP), and the anorexigenic peptides (appetite suppressing) proopiomelanocortin (POMC), and cocaine- and amphetamine-regulated transcript (CART). These neurones respond to peripheral metabolic hormones, including leptin, insulin, ghrelin and nutrients. POMC neurons project to second-order neurons in the hypothalamic paraventricular nucleus (PVN), the dorsomedial hypothalamus (DMH), the lateral hypothalamus (LH) and the ventromedial hypothalamus (VMH) (8).

### 1.2 The Anorexigenic Hypothalamic Pathway

Peripheral signalling from leptin and insulin suppresses appetite through the activation of POMC and CART (9, 10), leading to an increase in the anorexigenic neuropeptide α-melanocyte-stimulating hormone (α-MSH) that binds to the melanocortin-3 and -4 receptors (MC3R and MC4R) in the hypothalamic PVN and LH, reducing food intake and increased energy expenditure (11). This is mediated by efferent neuronal projections from the PVN and LH that synapse in extrahypothalamic brain regions within the locus coeruleus, leading to activation of the sympathetic nervous system, and regulation of parasympathetic tone *via* the vagus nerve through the dorsal motor nucleus of the vagus (DMV) resulting in an increase in energy expenditure and net catabolic effect. Insulin secretion is regulated by parasympathetic vagal-efferent signalling by promoting depolarization of pancreatic *β*-cells *via* M3-muscarinc receptors (12, 13). Vagus nerve–mediated acetylcholine also increases intracellular phospholipases within *β*-cells, increasing intracellular release of calcium, inducing insulin vesicular exocytosis and insulin secretion (14, 15). Parasympathetic nervous system innervation to the pancreas is also associated with increased *β*-cell proliferation and mass (16), suggesting that the insulin hypersecretion secondary to hypothalamic damage is likely to result from increased *β*-cell proliferation and mass as well as insulin hypersecretion. Early studies demonstrate that ablation of the VMH in rats results in the insulin hypersecretion which can be obviated by pancreatic vagotomy (17, 18). Thus, damage to the VMH and LH results in hyperinsulinemia and a net reduction in lipolysis and fat mass accumulation. In the energy replete state, anorexigenic pathways within the hypothalamus activated by increased insulin and leptin stimulate downstream sympathetic efferent signalling resulting in increased brown adipose tissue thermogenesis and energy expenditure in part by raising resting heart rate and mean arterial pressure (19). As beta-adrenergic signalling also promotes lipolysis (20) and limits insulin secretion (21), abrogation of sympathetic outflow from the hypothalamus secondary to hypothalamic damage will result in disinhibited insulin secretion, a reduction in lipolysis with fat mass accumulation and reduced energy expenditure.

### 1.3 The Hypothalamic Orexigenic Pathway

During fasting or energy deficit, neurons situated in the ARC stimulate feeding when they are activated by hormones such as ghrelin (12, 13) through the influence of NPY, AgRP and the neurotransmitter GABA on the PVN (5). AgRP downregulates the production of MC3R and MC4R, thereby preventing the anorexigenic effect of α-MSH on second-order neurones (22). AgRP/NPY neurons also directly inhibit POMC neurons *via* GABA at the level of the ARC (23). GABA release from AgRP/NPY projections to extrahypothalamic neurons, in the parabrachial nucleus, also plays a role in the stimulation of food intake (24). As well as stimulating feeding, activation of NPY results in energy conservation by reducing the metabolic activity of brown adipose tissue in a manner paradoxical to that seen with regulation of thermogenesis by POMC, by downregulation of sympathetic outflow from the locus coeruleus (25).

### 1.4 The Role of Insulin and Leptin in the Control of Feeding, and Energy Homeostasis

Centrally, POMC and AgRP/NPY neurons express receptors for insulin and leptin, indicating that these hormones play a key role in energy homeostasis and food intake. The adipocyte derived hormone leptin circulates at plasma levels directly correlated to adiposity (26) and plays a key role in energy homeostasis as a negative feedback regulator of adiposity by limiting energy intake and supporting energy expenditure thus preventing weight gain (27). Thus, during periods of starvation during which time fat mass is reduced, leptin is reduced in-turn promoting increased food intake and fat accumulation (28); conversely disruption of leptin signalling promotes hyperphagia and rapid weight gain (29). In the mediobasal hypothalamus, leptin activates POMC whilst directly inhibiting AgRP and NPY neurons with a net effect of increasing energy expenditure and decreasing food intake (30). In addition to this, in the dorsomedial hypothalamus, leptin promotes increased energy expenditure through activation of brown adipose tissue which results in a reduction in body weight that is independent of food intake (31).

**Figure 1:**
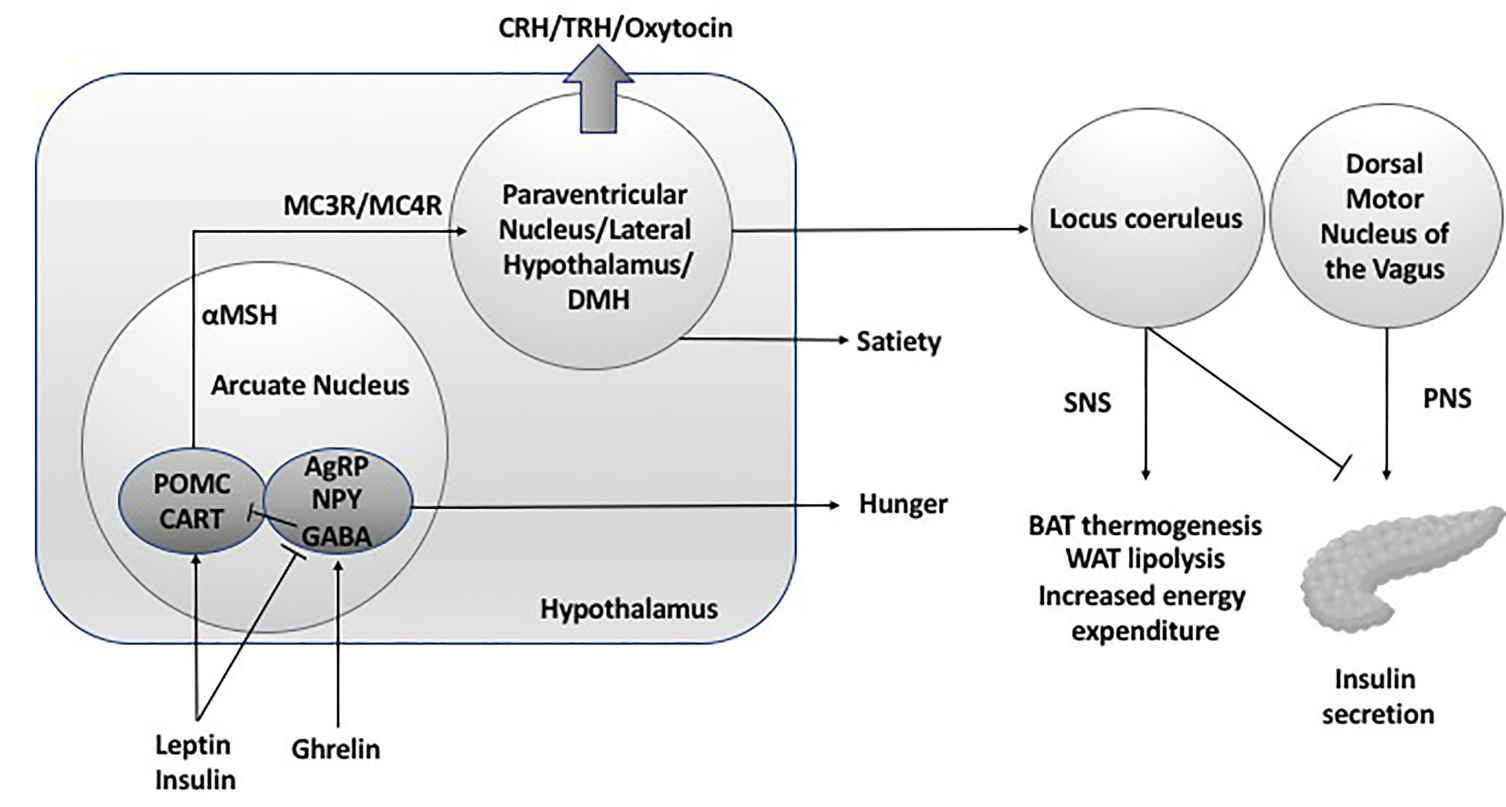
Hypothalamic neuroendocrine regulation of energy expenditure and feeding. DMH, dorsal medial hypothalamus; SNS, sympathetic nervous system; PNS, parasympathetic nervous system; POMC, pro-opiomelanocortin C; CART, cocaine and amphetamine related transcript; AgRP, Agouti related peptide; NPY, neuropeptide Y; GABA, gamma aminobutyric acid; MC3R, melanocortin 3 receptor; MC4R, melanocortin 4 receptor; BAT, brown adipose tissue; WAT, white adipose tissue; CRH, corticotroph releasing hormone; TRH, thyrotroph releasing hormone.

Insulin is secreted from pancreatic β-cells upon nutrient ingestion and plays an important role in the peripheral regulation of energy and glucose homeostasis by peripheral glucose metabolism through the suppression hepatic glucose production *via* direct action on hepatic insulin receptors. Centrally, disruption of insulin receptors in the hypothalamus causes obesity, insulin resistance, hyperphagia, and hyperleptinemia in mice (32) and when insulin was injected into the cerebral ventricles of baboons in a study over 40 years ago, food intake and endogenous glucose production were both suppressed (9) underpinning the central role of insulin in energy homeostasis. The centrally mediated action of insulin has since been extensively reviewed in the last few years. At the level of the hypothalamus, insulin acts to suppress food intake, promote peripheral lipogenesis, inhibit hepatic glucose production and promote brown adipose tissue thermogenesis. These centrally mediated actions of insulin are fundamentally mediated through the excitation of POMC neurons and the concomitant suppression of AgRP and NPY neurons (33–35). Hypothalamic pathways regulating energy expenditure and feeding are detailed in **Figure 1**.

Given the fundamental role of the hypothalamus in energy homeostasis and appetite regulation, it follows that damage to the hypothalamus results in dysregulation of satiety and energy expenditure, leading to hyperphagia and rapid weight gain, reduced sympathetic tonicity and insulin hypersecretion. Thus, this provides multiple target areas for pharmacotherapeutic intervention to reduce weight gain and fat mass in patients with hypothalamic obesity.

## 2 Weight Gain in Children With Acquired Hypothalamic Damage

Obesity is a well-recognized and common complication of hypothalamic damage either as a result of tumour invasion of, or treatment to, the hypothalamic regions critical to energy regulation. Imaging studies have demonstrated a direct correlation between the extent of hypothalamic damage and presentation of obesity (36, 37). In patients with craniopharyngioma, hypothalamic obesity occurs in up to 50% of patients (38–41) and up to twenty percent of patients with craniopharyngioma are obese at diagnosis (39–41), and this weight gain is irrespective of pituitary deficiency secondary to damage to the hypothalamic-pituitary axis. Higher preoperative BMI, radical tumour resection, larger preoperative tumour size, hypothalamic tumour invasion, adamantinomatous subtype, and familial predisposition to obesity are cited as factors that increase the risk of hypothalamic obesity (37, 42, 43). Rapid weight gain usually occurs within the first 3 years and often within the first year following surgical intervention, with surgical intervention increasing the prevalence of obesity in this patient group (38, 43).

Leptin and insulin levels are higher in patients with hypothalamic obesity suggesting that the normal mechanism by which leptin and insulin mediate satiety and play a central role in energy homeostasis is impaired, leading to uncontrolled and rapid gain in fat mass secondary to impaired satiety and disruption of energy homeostasis (44, 45). Assessment of dietary intake, energy expenditure and basal metabolic rate (BMR) in patients with craniopharyngioma report similar food intake to healthy controls, but instead demonstrate that reduced physical activity, energy expenditure and BMR may be central to rapid weight gain (45–47). Decrease in energy expenditure and basal metabolic rate is likely to be associated with a reduction in sympathetic tone as a direct result of hypothalamic damage. Evidence supporting this theory includes a reduction in the excretion of the urinary catecholamine metabolites homovanillic acid and vanillylmandelic acid that directly correlates with BMI (48), and a reduction in the 24-hour urinary excretion of adrenaline that is independent of adrenal insufficiency from pituitary damage (49).

In addition to the role that the hypothalamus plays in energy regulation, patients with hypothalamic obesity suffer with other co-morbidities that include endocrine dysfunction, sleep disturbance, visual dysfunction and neurological sequalae, underlying the fundamental role that the hypothalamus plays in physiological control, and the resultant damage caused by suprasellar tumours due to the intimate co-location of the hypothalamus with other structures such as that optic chiasm and cavernous sinus. The pituitary gland is dependent on hypothalamic signals that are frequently disrupted from hypothalamic damage, that affects secretion of growth hormone, gonadotropins, adrenocorticotrophic hormone (ACTH) and thyroid stimulating hormone (TSH). At the time of diagnosis up to 90% of patients with craniopharyngioma are reported to have at least one pituitary hormone deficiency (39, 40, 50). Thus, correction of pituitary hormone deficiency is key to the management of patients with suprasellar tumours. In particular, deficiency in growth hormone, and hypothyroidism secondary to TSH deficiency from the pituitary or thyrotropin releasing hormone from the hypothalamus (TRH), can compound excessive weight gain if left untreated, and treatment of these hormone deficiencies results in reduction in weight (51). Moreover, change in behaviour secondary to estrogen or testosterone deficiency due to hypogonadotrophic hypogonadism can lead to low mood and result in a reduction in physical activity thus compounding weight gain.

The hypothalamus is also a central regulator of sleep and arousal. The impact of hypothalamic lesions leading to sleep disruption was reported nearly 100 years ago (52). Hypothalamic damage results in disturbances in sleep-wake regulation with alterations in the circadian rhythm, sleep fragmentation, and increased daytime somnolence (53, 54). Polysomnography in children with craniopharyngioma demonstrates sleep patterns consistent hypersomnia and secondary narcolepsy (55, 56). This can be compounded by obstructive sleep apnoea secondary to obesity, leading to daytime somnolence secondary to poor sleep quality at night (57). Given that sleep is considered to be a period of energy conservation, hypersomnia in patients with hypothalamic damage can result in a reduction in energy expenditure (58). Concomitantly, although sleep disruption results in a rise in energy expenditure, energy consumption exceeds this rise resulting in a net weight gain (59). This is part is due to appetite dysregulation secondary to an increase in ghrelin and reduction in leptin (60), poor diet quality, disruption in the timing of eating, and a change in eating behaviours that promotes intake of higher calorific foods and emotional eating (61).

Due to the intimate co-location of the hypothalamus, pituitary and optic chiasm, visual disturbance from suprasellar tumours is relatively common at presentation, with visual disturbance identified in more than 50% of patients who present with craniopharyngioma (39). Moreover, due to the co-location of the hypothalamus to structures within the cavernous sinus, craniopharyngiomas can lead to other neurological sequalae including epilepsy, cranial nerve dysfunction and cerebrovascular events which increase in frequency with larger tumours (62). Any of these deficits either in isolation or in combination has the potential to impact on the ability to partake in physical activity, and thus increase the risk of obesity.

## 3 Current Approaches to Managing Hypothalamic Obesity

### 3.1 Surgery and Radiotherapy for Hypothalamic Lesions

Craniopharyngiomas present a surgical challenge because of their proximity to the optic nerves optic, pituitary, hypothalamus, circle of Willis, brain stem, and temporal lobes. To minimize the risk of hypothalamic obesity, treatment strategies have become more conservative in an attempt to preserve normal hypothalamic function. Gross total resection results in higher rates of hypothalamic obesity, panhypopitutarism and limited improvement in 5-year progression-free survival (PFS) (63). Gross total resection is now used much less commonly and total tumour resection should be limited to tumours of less than 4 cm that can be easily removed without sequalae. The aim of surgical intervention should be to relieve tumour-associated compression symptoms, preserve or improve vision and hypothalamo-pituitary function, and minimize tumor recurrence through adjunct radiotherapy (64). Subtotal resection in isolation is associated with significantly inferior 5-year PFS compared with conservative surgery followed by adjuvant radiation (65). The approach of using subtotal resection followed by radiotherapy is of particular importance in patients with lesions within the hypothalamus as they have decreased 10-year overall survival and a significant risk of poor psychosocial and quality of life (66). More recently, surgical and radiotherapeutic approaches have evolved in an attempt to minimise treatment related damage. Current techniques include fractionated three-dimensional conformal radiotherapy, intensity modulated radiotherapy (IMRT), fractionated stereotactic radiotherapy (FSRT) and proton beam therapy. IMRT is an advanced mode of high-precision radiotherapy that delivers precise radiation doses to conform more precisely to the three-dimensional shape of a tumour by modulating the intensity of the radiation beam in multiple small volumes, thus limiting radiation scatter to surrounding unaffected structures by up to 45% (67). Proton beam therapy is now used preferentially in the treatment of craniopharyngiomas due to the ability to limit the distribution and intensity of the radiation dose, in-turn reducing the risk of complications and secondary cancer, whilst improving target conformity (68, 69), with some case series reporting a 100% 5-year control rate (70–72). Extension beyond tumour margins is now limited to 5mm to prevent the extension of radiation to normal surrounding structures (73). Collectively, these modern approaches to tumour radiation result in tumour control between 80-90% (74). Hypothalamic obesity has been reported in only 25% of children receiving IMRT or PBT (75), but in general hypothalamic obesity is under-reported and case-series are most-commonly retrospective. Prospective studies are required to determine whether a more conservative surgical approach together with newer radiotherapy modalities can reduce the incidence of post-interventional hypothalamic obesity.

### 3.2 Pharmacotherapeutics

The recent advances in our understanding of the centrally mediated pathways relevant to energy and appetite regulation have resulted in a targeted pharmacological approach in an attempt to bypass damaged hypothalamic pathways.

#### 3.2.1 Targeting Insulin Hypersecretion

Damage to the VMH, PVN and LH results in a rise in vagal tone resulting in insulin hypersecretion, promoting energy deposition into the adipocyte and increased fat mass. The anorexigenic effect of insulin *via* afferent hypothalamic pathways may either be blunted or ablated by hypothalamic damage (9, 10). Pragmatically, carbohydrate restriction would appear to be the simplest way to reduce insulin secretion, although evidence to this approach is generally lacking and in general food intake does not appear to be significantly altered in hypothalamic obesity (45–47). Diazoxide is an inhibitor of glucose-stimulated insulin release and has been successfully used to lower insulin secretion in children with congenital hyperinsulinaemia. Diazoxide therapy in patients with hypothalamic obesity has however, been unsuccessful. In a study of 40 children, 18 of whom were randomized to a treatment arm using diazoxide (4mg/kg/day), there was no difference in weight when compared with the placebo group. More worryingly, 3 patients developed diabetes mellitus and plasma glucose was significantly higher in the treatment group (76).

In contrast, the combination of metformin and diazoxide has shown slightly more promising results in slowing weight gain (albeit not leading to weight loss). Metformin improves insulin sensitivity and decreases hepatic gluconeogenesis and intestinal glucose absorption. In a prospective open-label 6-month pilot treatment trial in 9 obese subjects with craniopharyngioma the administration of diazoxide (2 mg/kg/day) and metformin (1000 mg twice daily) resulted in an overall reduction in weight gain over a 6-month period compared with the weight gain observed over the 6-month period prior to the intervention. However, 2 patients were withdrawn due to vomiting and peripheral edema. This study is notably limited by the small number of participants and the lack of a comparator group, by instead assuming that weight gain would be uniformly similar during the pre-treatment and treatment phases (77).

Combination therapy of metformin and fenofibrate in hypothalamic obesity secondary to craniopharyngioma has again yielded disappointing results. Fenofibrate activates peroxisome proliferator-activated receptors alpha (PPAR-alpha), which upregulates lipoprotein lipase, induces high-density lipoprotein synthesis, and decreases liver production of apolipoprotein C and improves insulin sensitivity (78). Despite no significant improvement in weight gain, the fenofibrate/metformin combination therapy resulted in a reduction in dyslipidemia and improved insulin sensitivity in the treatment group (79).

Octreotide is a somatostatin analogue that improves insulin sensitivity (80). An initial pilot study in 1999 in eight paediatric patients demonstrated a reduction in body mass index that correlated with reduced insulin secretion and plasma leptin. However, a subsequent double-blind study enrolled 20 patients with hypothalamic obesity (13 subjects had craniopharyngioma, 4 subjects had hypothalamic astrocytoma, 1 had pineal germinoma, and 2 subjects had acute lymphoblastic leukaemia and received 24 Gy of cranial irradiation). Two patients were discontinued, one on octreatide who had recurrence of their craniopharyngioma, and the other on placebo developed diabetic hyperosmolar nonketotic coma. The 9 patients who were treated with octreotide showed a statistically significant decrease in BMI (i.e., –0.2 ± 0.2 on treatment versus +2.2 ± 0.5 on placebo) and improvement in insulin secretion that correlated with quality of life. Complications included diarrhea and abdominal discomfort in all those treated with octreotide which eventually subsided, and 4 patients developed gallbladder sludge or true cholelithiasis on ultrasound. Two patients developed impaired glucose tolerance, and 1 patient developed type 2 diabetes during the open-label extension, although this latter patient already exhibited acanthosis nigricans prior to treatment (81). A subsequent phase 4 study of octreotide depot compared with saline control in paediatric hypothalamic obesity patients led by Novartis (ClinicalTrials.gov Identifier: NCT00171613) commenced in February 2005 with patients receiving sandostatin (octreotide) LAR (SAS-LAR) depot for 6 months. After 6 months of treatment with SAS-LAR or placebo, no difference in change from baseline in BMI was seen between treatment group (0.1 kg/m^2^ in the SAS-LAR-treated group versus 0.0 kg/m^2^ in the placebo-treated group, p=0.74) (82). No further trials using octreotide therapy have been published since.

### 3.3 Pharmacological Intervention on Energy Expenditure and Appetite

Given the evidence demonstrating a reduction in energy expenditure and BMR in patients with hypothalamic obesity (45–47), therapies that increase energy expenditure have been trialled to reduce BMI. CNS stimulants such as dextroamphetamine (83), sibutramine (84, 85) and a combination of caffeine and ephedrine (86) have been shown to reduce appetite and promote weight loss, albeit that sibutramine has since been withdrawn due to concerns over cardiovascular complications (84).

The rationale for using ephedrine in the treatment of hypothalamic obesity is based upon the reduction in sympathetic tone seen in these patients. Ephedrine is a sympathomimetic amine that activates adrenergic receptors, increasing heart rate and blood pressure, improving energy expenditure and increasing brown adipose tissue activity (87, 88). Ephedrine activates adrenergic α and β-receptors as well as inhibiting noradrenaline reuptake, and increasing the release of noradrenaline from vesicles in nerve cells. Caffeine affects peripheral metabolism through alterations in sympathetic nervous system activity (89) and by influencing peripheral metabolic targets directly through inhibition of cAMP phosphodiesterase or adenosine receptors or by activation of AMP-kinase (90). Three patients treated with a combination of caffeine and ephedrine showed an initial 8-18% reduction in weight, with 2 out of 3 showing sustained weight loss for 2 and 6 years respectively, and the other returning to the baseline weight (91). However, a meta-analysis of ephedrine with and without caffeine for weight loss and improving athletic performance demonstrated a 2.2 to 3.6 -fold increase in the odds of psychiatric, autonomic, or gastrointestinal symptoms and heart palpitations (86), thus underpinning the need to conduct a randomised placebo-controlled trial of caffeine and ephedrine in patients with hypothalamic obesity to monitor for treatment effect and complications.

The centrally mediated appetite suppressant side effect seen in patients with ADHD and narcolepsy treated with dextroamphetamine (92) suggests that a similar effect may be observed in patients with hypothalamic obesity. Dextroamphetamine mediates central anorexigenic control by direct modification of cerebral satiety signalling through stimulation of cocaine-amphetamine regulated transcript (CART) production in the ventromedial hypothalamus. Dextroamphetamine is also a potent sympathomimetic agent leading to an increase in α- and β-adrenergic tone (93), which may counteract the reduce sympathetic tone observed in hypothalamic obesity patients. Increased activity of the mesolimbic reward pathway and elevated extracellular dopamine in the nucleus accumbens following administration of dextroamphetamine may also rectify the compulsion to eat excessively (94). This action may be consistent with functional magnetic resonance imaging studies that demonstrate a trend towards higher activation in cerebral regions associated with hedonic appetite including the insula, nucleus accumbens, and medial orbitofrontal cortex observed in craniopharyngioma patients with hypothalamic obesity (95). In a study of 12 patients with hypothalamic obesity treated with low dose dextroamphetamine (5mg twice daily), 10 of the patients either lost weight or demonstrated weight stabilization and daytime somnolence improved in 11 patients (96). A similar effect of weight stabilization was observed in the first year in a case series of 7 patients treated with an upward dose titration regimen of dextroamphetamine commencing at 5 mg per day up to 20mg per day, although the results of the second year of dextroamphetamine treatment were heterogenous with several patients continuing to reduce weight, while others demonstrated significant gains in BMI z-score (97). In an earlier study of 5 children with hypothalamic obesity secondary to surgical intervention for craniopharyngioma, weight gain stabilized and significant improvements were observed in attention and activity following dextroamphetamine treatment (98). Longer-term studies are required to determine whether weight loss or stabilization is sustained by using dextroamphetamine in the treatment of hypothalamic obesity, to understand the mechanism by which dextroamphetamine is exerting an effect (e.g. as a sympathomimetic agent) and the potential for longer-term side effects.

In a small number (n=3), tri-iodothyroine monotherapy (T3) has also been trialled in patients with hypothalamic obesity, with a sustained reduction in weight observed over a 2-year period and with improvement in daytime somnolence (99). Only one additional study of T3 supplementation has been conducted since in a patient with hypothalamic obesity, by which levothyroxine therapy was switched to T3 monotherapy, with results demonstrating no change in sympathetic and metabolic BAT activity, energy expenditure, or BMI during T3 treatment (100). Thus, to date, only 4 patients have undergone treatment with T3 monotherapy with conflicting results. Given the ubiquitous impact on metabolic function mediated by thyroid hormones (101), further work is needed in this area to elucidate whether a select group of patients with hypothalamic obesity may respond with weight loss to T3 monotherapy.

## 4 Novel Therapeutic Approaches – Future Therapies for Hypothalamic Obesity

### 4.1 GLP1-Analogues

An alternative approach to appetite regulation in patients with established hypothalamic obesity is to target areas of the brain that control satiety that are not impacted by hypothalamic damage. The quantity of food consumed is regulated by the nucleus tractus solitarus (NTS) located in the dorsomedial medulla and is controlled by gut mediated vagal afferents influenced by gut peptides including GLP1 and CCK (102, 103). Leptin appears to potentiate this effect by directly and indirectly enhancing the response of the NTS to gut peptides and leptin is increased in patients with hypothalamic obesity (6, 27, 104, 105). GLP1 receptor analogues (GLP1A) may therefore potentiate NTS sensitivity to GLP1 thus reducing the frequency and quantity of food consumed, leading to weight loss. In a rat model recapitulating the key features of hypothalamic obesity, the use of the GLP1A exendin-4 resulted in a significant reduction in food intake and weight compared to those treated with saline (106). However, results in adult studies to date have been variable with one adult study demonstrating sustained weight loss over 6-51 months in 9 patients with hypothalamic obesity and type-2 diabetes following treatment with a GLP1A (107), with a further 8 patients treated with twice daily exanetide for 1 year showing no significant weight loss, but no weight gain (108). The first study of children given 2 mg exenatide weekly for a 12-month period again showed no significant impact on weight or BMI, albeit one patient demonstrated a BMI SDS reduction of -0.33 after 12 months (109). In contrast, a recent randomized, multicentre, double-blind, placebo-controlled trial was conducted in 10- to 25-year-olds with hypothalamic injury following intracranial tumour and hypothalamic obesity. Participants were randomised to once-weekly subcutaneous injections of exenatide 2 mg or placebo for 36 weeks. Whilst there was no significant reduction in BMI in the treatment group compared to controls, there was a reduction in total body fat mass as measured by DXA in and stabilization of waist circumference in 50% of the treatment group, compared to an increase in waist circumference and fat mass in controls. Exanetide was generally well tolerated with the majority of side effects being related to gastrointestinal disturbance (110). The limited impact in response to GLP1A therapy on food consumption and weight loss in patients with hypothalamic obesity, may reflect a disruption of pathways involved in the complex interaction between the hypothalamus and other areas of the brain involved in hunger, satiety and hedonic appetite, that are disrupted and thus unresponsive to therapies that are aimed at targeting areas of the brain distant from the hypothalamus. Moreover, a select group of patients with limited hypothalamic damage may respond better to GLP1A, whilst others with more extensive hypothalamic damage fail to respond to the same therapy. However, contrary to this theory, stratification by MRI prior to enrolment into a trial of GLP1A to assess the degree of hypothalamic damage using an integrative hypothalamic lesion score (HLS), demonstrated that patients with more extensive hypothalamic damage with involvement of the mammillary body show greater reductions in adiposity following GLP1A treatment. The authors speculated that disruption of hypothalamic pathways involved in appetite and energy homeostasis may result in alterations in other pathways such as GLP1-mediated signalling in the brainstem, which remain intact in patients with hypothalamic obesity (111). The relevance of the mammillary body damage in this study is poorly understood in relation to GLP1A responsiveness and suggests that the mammillary body damage may act as a proxy for more extensive hypothalamic damage, or that the mammillary body has a functional role in feeding behaviour *via* interaction with the hippocampus (112). Future studies in patients treated with exanetide may thus benefit from additional stratification based on the degree of hypothalamic damage.

### 4.2 Oxytocin

Hypothalamic PVN neurons secrete neuropeptides that have a net catabolic action, including corticotrophin-releasing hormone, thyrotropin-releasing hormone, somatostatin, vasopressin and oxytocin. In the hypothalamic PVN, oxytocin and other neurons tonically inhibit feeding and, during energy deficit, are inhibited by orexigenic input from the ARC, thereby stimulating feeding (11), leading the concept that oxytocin supplementation may promote weight loss in hypothalamic obesity. In 2018, administration of intranasal oxytocin was given for 10 weeks, followed by a combination of intranasal oxytocin and naloxone for 38 weeks in a 13-year-old male with confirmed hypothalamic obesity and hyperphagia following resection of a craniopharyngioma. The observed reduction in BMI and hyperphagia following oxytocin monotherapy has paved the way for future trials (113). Oxytocin is a neuropeptide produced in the hypothalamic PVN and supraoptic nucleus (SON) and modulates food intake and behaviour. The anorexigenic effect of oxytocin results from the extension of oxytocin neurons from the PVN and SON to other distal brain targets involved with appetite regulation (114). For example, the medial NTS receives oxytocinergic input that potentiates the effect of afferent gastrointestinal inputs resulting in a reduction in food intake (115). Damage to the hypothalamus from craniopharyngiomas and other tumours will result in disruption of POMC activation of PVH magnocellular oxytocin neurons and the release of oxytocin *via* α-melanocyte stimulating hormone and will thus abrogate the anorexigenic effect of oxytocin (116, 117). Moreover, VMH oxytocin administration has been shown to increase short-term energy expenditure and spontaneous physical activity (118, 119). Thus, oxytocin replacement may stimulate anorexigenic pathways that have been downregulated through hypothalamic damage and may play a role in upregulating pathways involved in energy expenditure. In patients with Prader-Willi syndrome (PWS), a syndrome associated with congenital hypothalamic damage, hypothalamic obesity and hyperphagia, intranasal oxytocin leads to a reduction in appetite drive, and improvements in socialization, anxiety, and repetitive behaviours. However, further, long-term studies with a larger population of participants are required to determine whether these effects are sustained (120, 121). Results from a placebo-controlled trial using Syntocinon (synthetic oxytocin) administered three times a day with meals in patients aged 10-21 years with hypothalamic obesity are awaited (ClinicalTrials.gov Identifier: NCT02849743).

### 4.3 Methionine Aminopeptidase Inhibitors (MetAP2)

Methionine Aminopeptidase Inhibitors are enzymes responsible for the removal of methionine from the amino-terminus of newly synthesized proteins (122). Increased expression of *METAP2* gene is associated with various forms of cancer (123), and thus methionine aminopeptidase 2 inhibitors (MetAP2) were initially developed for treatment of cancer by preventing tumour proliferation (124). However, MetAP2 therapy also resulted in weight loss with increased adiponectin and decreased leptin, leading to decreased lipogenesis, increased fat oxidation, and increased lipolysis (125). The anti-obesity efficacy of MetAP2 inhibitors has been demonstrated in animal models of obesity and in humans at low doses that do not affect angiogenesis (126, 127). The precise mechanism for the anti-obesity effect of MetAP2 inhibitors is not clear but the ability of MetAP2 to suppress activity of extracellular signal regulated kinases 1 and 2 (ERK1/2) represents one of the key mechanisms for the observed anti-obesity effect (128). In the hypothalamic obesity mouse model, administration of the methionine aminopeptidase 2-inhibitor beloranib resulted in a reduction in body weight primarily related to a 30% reduction in food intake with improved glucose tolerance, reduced plasma insulin, and increased circulating levels of α-melanocyte stimulating hormone (129). A subsequent randomized, double-blind, placebo-controlled trial of 26-week beloranib therapy in patients with PWS demonstrated a reduction in food-seeking behavior and hyperphagia as well as weight loss secondary to an overall 85% reduction in fat mass, but the trial was stopped early due to an increase in serious thromboembolic events (2 fatal events of pulmonary embolism and 2 events of deep vein thrombosis) and mental health issues compared with placebo (130). In a Phase 2a, double-blind, placebo-controlled study of 14 adults with hypothalamic obesity, randomized to receive beloranib 1.8 mg or placebo subcutaneously twice weekly for 4 weeks with an optional 4-week open-label extension, patients in the treatment group lost weight that was sustained during the 4-week extension. No major adverse events were reported but the trial was short in duration (131). In the future, specific MetAP2 inhibitors developed to reduce the risk of thromboembolism may hold promise in the treatment of hypothalamic obesity.

### 4.4 Tesomet (Tesofensine and Metoprolol)

Tesomet is an investigational fixed-dose combination therapy of tesofensine (a triple monoamine reuptake inhibitor) and metoprolol (a beta-1 selective blocker). Tesofensine acts on the brain to block reabsorption of three monoamine neurotransmitters - serotonin, noradrenaline and dopamine (132, 133), leading to a reduction in food cravings and subsequent reduction in weight. Tesofensine was originally developed to treat Alzheimers and Parkinson’s disease, but it was found to be ineffective and the unintended side effect of weight loss was observed in multiple clinical trials (134, 135). A phase II study of tesofensine in obese, nondiabetic subjects in 2008 demonstrated weight loss that was associated with increased doses of tesofensine, and beneficial effects on blood triglycerides, cholesterol, insulin and HbA1c were observed. Tesofensine has been combined with metoprolol to counteract previously observed side effects of tachycardia and hypertension at higher doses (136) thought to be related to an increase in sympathetic activity, which in fact may be of benefit to counteract the reduced sympathetic tone in patients with hypothalamic obesity (137). An ongoing phase 2 trial to evaluate the safety and efficacy of Tesomet in subjects 16 years of age or older with hypothalamic obesity is due to complete in May 2024 (ClinicalTrials.gov Identifier: NCT05147415).

### 4.5 Selective MC4 Receptor Agonists

**Figure 2:**
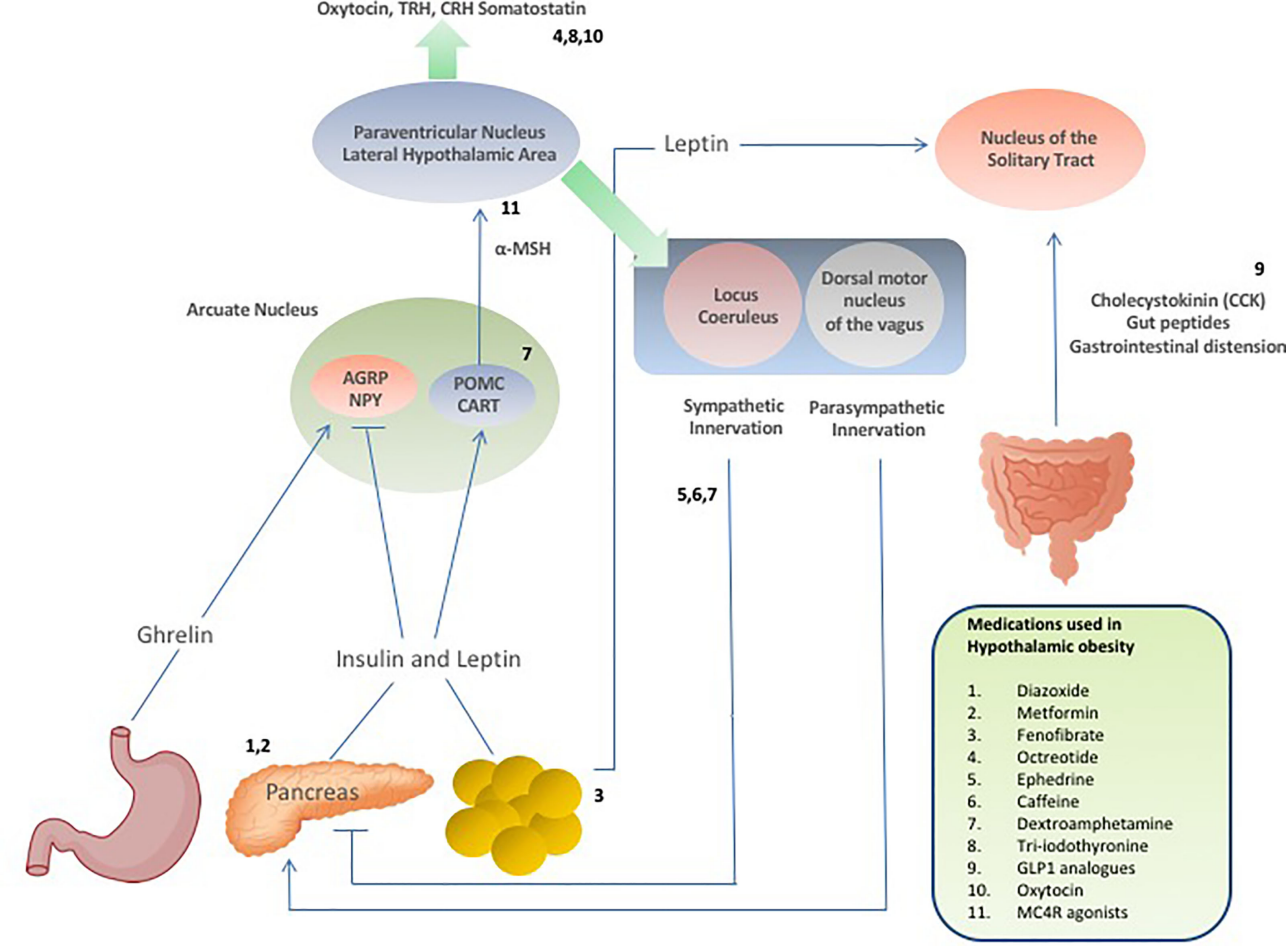
Schematic demonstrating hormonal and neuropeptide cerebral regulation of energy homeostasis and appetite and the selective sites of targeted drug action to suppress appetite and promote weight loss in patients with hypothalamic obesity.

Setmelanotide is a selective MC4 receptor agonist licensed for 6 years and older with obesity due to pro-opiomelanocortin deficiency, proprotein subtilisin/kexin type 1 (PCSK1) deficiency, and leptin receptor (LEPR) deficiency confirmed by genetic testing and is currently being trialled in other rare genetic disorders associated with obesity including Bardet-Biedl Syndrome and Alström Syndrome (138, 139). Setmelanotide stimulates MC4 receptors in the PVN and LH to activate anorexigenic pathways (140, 141). First year results of setmelanotide in patients with Bardet-Bield syndrome are promising, with weight loss and appetite suppression reported with no major adverse events (141). Results are awaited from a phase 2, open-label 20-week study to evaluate the safety and efficacy of Setmelanotide in subjects with hypothalamic obesity (ClinicalTrials.gov Identifier: NCT04725240), although drug response may be minimal due to potential damage of the PVN in these patients. A summary of the pharmacological interventions for hypothalamic obesity is provided in **Table 1**, and **Figure 2** provides a schematic representation of the selective sites of targeted pharmacological intervention to suppress appetite and promote weight loss in patients with hypothalamic obesity.

**Table 1:** Targeted action of pharmacological therapies in hypothalamic obesity.

Drug	Mechanism/target
Diazoxide	To overcome insulin hypersecretion from hypothalamic damage by reducing insulin secretion from the pancreas
Metformin	To overcome insulin hypersecretion from hypothalamic damage by improving insulin sensitivity and decreasing hepatic gluconeogenesis and intestinal glucose absorption
Fenofibrate	To overcome insulin hypersecretion from hypothalamic damage by improving insulin sensitivity through lipolysis
Octreotide	To overcome insulin hypersecretion from hypothalamic damage by improving insulin sensitivity
Ephedrine	Sympathomimetic amine that activates adrenergic receptors, increasing heart rate and blood pressure, improving energy expenditure and increasing brown adipose tissue activity to overcome reduced sympathetic tone on hypothalamic obesity
Caffeine	Sympathetic nervous system stimulant to overcome reduced sympathetic tone on hypothalamic obesity
Dextroamphetamine	Mediates central anorexigenic control by direct modification of cerebral satiety signalling through stimulation of cocaine-amphetamine regulated transcript (CART) production in the ventromedial hypothalamus, to induce appetite suppression
Tri-iodothyronine	Increase metabolic rate and energy expenditure to overcome the negative impact on energy expenditure in hypothalamic obesity
GLP1 analogues	Potentiate nucleus tractus solitarus in the brain stem sensitivity to GLP1 reducing the frequency and quantity of food consumed, leading to weight loss in hypothalamic obesity
Oxytocin	Induces appetite suppression and feeding reward behavior in the hypothalamus and other parts of the brain
Methionine Aminopeptidase Inhibitors (MetAP2)	Direct target unknown. MetAP2 therapy results in weight loss with increased adiponectin and decreased leptin, leading to decreased lipogenesis, increased fat oxidation, and increased lipolysis
Tesomet (tesofensine and metoprolol)	Tesofensine acts on the brain to block reabsorption of three monoamine neurotransmitters - serotonin, noradrenaline and dopamine leading to a reduction in food cravings and subsequent reduction in weight
Selective MC4R agonists	Directly induce appetite suppression at the level of the hypothalamus to mimic the action of prop-opiomelanocortin C

## 5 Surgical Intervention to Reduce Calorific Intake

A number of bariatric surgical approaches have been trialled in patients with hypothalamic obesity including Roux-en-Y Gastric Bypass (RYGB), Laproscopic Gastric Banding (LAGB) and sleeve gastrectomy (SG). In a number of patients, weight loss is relatively short-lived with patients returning to pre-surgical weights in studies with the longest follow-up duration, potentially reflecting the multi-faceted challenges of hypothalamic obesity that cannot be rectified by surgery, including reduced BMR and energy expenditure, low mood and increased daytime somnolence (142). The majority of studies reporting on bariatric surgery in patients with hypothalamic obesity include small number of patients. The largest study cohort reported includes 9 patients who underwent either LAGB (n = 6), SG (n = 4), and/or gastric bypass (n = 2). Two of these patients received SG after initial LAGB surgery, and one patient underwent gastric bypass after LAGB. No weight change occurred after LAGB or SG, but two patients who underwent gastric bypass surgery experienced weight loss after a mean follow-up period of 3 years (143). In a systematic review, van Iersel et al. report on 10 studies of bariatric surgery in patients with hypothalamic obesity. Of the studies that report on responders and non-responders to intervention, 19 of 24 patients experienced weight loss after bariatric surgery. The percentage of responders was 0.0% (0 of 4) for LAGB, 100.0% (6 of 6) for SG, and 92.9% (13 of 14) for gastric bypass surgery, although two patients regained weight after SG and RYGB (142).

To reduce insulin hypersecretion, truncal vagotomy has been reported in one patient resulting in 30 kg weight loss and reduced insulin secretion with delay in gastric emptying and foul smelling eructations as a side-effect (144). Other surgical approaches include the implantation of deep brain stimulation electrodes in a 19-year-old female to stimulate the nucleus accumbens, an approach that has been previously used in the treatment of morbid obesity (145, 146). DBS stimulation to the patient’s nucleus accumbens resulted in a sustained weight reduction and improved symptoms of hyperphagia after 14 months (147).

## 6 Conclusion

The challenge in achieving weight loss, modification in eating behaviour and improvement in energy expenditure in children and adults with acquired hypothalamic obesity reflects the complexity of hypothalamic control of energy homeostasis and feeding. Whist the hypothalamus is central to energy regulation and feeding, neuronal pathways to other parts of the brain are complex and extensive. In the last decade our understanding of central neuronal circuits that regulate feeding and satiety has led to the development of novel therapies capable of targeting afferent or efferent neuronal pathways that improve energy expenditure, modify insulin secretion and responsiveness, and upregulate anorexigenic and down regulate orexigenic neuropeptide pathways (148). The varied response to therapies in patients with hypothalamic obesity across studies may relate to the extent of hypothalamic damage, and future clinical trials will require enough patients to allow stratification to determine treatment response according to the location and extent of hypothalamic damage. Trials of this magnitude will require multinational collaboration to enroll enough patients to achieve relevant stratification. As our understanding of neuronal circuits controlling appetite and energy homeostasis outside of the hypothalamus increases, therapies should be developed that aim to circumvent damaged neuronal pathways, to target areas of the brain to modify eating behaviour and energy expenditure to promote weight loss. Given the thermogenic potential of brown adipose tissue, stimulating brown adipose tissue by novel targeted therapies may yield benefit in improving energy expenditure in patients with hypothalamic obesity. Trials using combinations of pharmacotherapeutic agents may yield more promising results but need to be considered carefully in the context of drug interactions and potential side effects. Combination therapies should potentially target one of the 6 domains of therapy recently proposed - hyperphagia, decreased energy expenditure, hyperinsulinemia, hypopituitarism, sleep disturbances, and psychosocial disorders (142). Bariatric surgical intervention may hold some promise in the treatment of hypothalamic obesity, but study numbers are small, and weight loss is potentially short-lived. The role of deep brain stimulation may also merit further consideration. Future trials should also consider a multidisciplinary approach by which novel therapies are combined with specific dietary intervention and exercise regimes (142, 149, 150). Ultimately, there is a significant need to act early to limit or reverse the rapid weight gain in patients with hypothalamic obesity to prevent the multiple associated physical and psychological co-morbidities that lead to early mortality in this patient population.

## Author Contributions

The author confirms being the sole contributor of this work and has approved it for publication.

## Conflict of Interest

The author declares that the research was conducted in the absence of any commercial or financial relationships that could be construed as a potential conflict of interest.

## Publisher’s Note

All claims expressed in this article are solely those of the authors and do not necessarily represent those of their affiliated organizations, or those of the publisher, the editors and the reviewers. Any product that may be evaluated in this article, or claim that may be made by its manufacturer, is not guaranteed or endorsed by the publisher.
